# Challenges and Management Outcomes of Osteoarticular Infections in Adult Sickle Cell Disease Patients

**DOI:** 10.3390/jcm14238542

**Published:** 2025-12-02

**Authors:** Mashael M. Alhajri, Marwan Jabr Alwazzeh, Ghayah Almulhim, Ahmed Alsahlawi, Mohammed A. Alharbi, Faleh Alotaibi, Bader Salamah Alanazi, Ahmed Salamah Alzahrani, Fahad Aljabbari

**Affiliations:** 1 Infectious Diseases Division, Department of Internal Medicine, King Fahad Hospital of the University, Faculty of Medicine, Imam Abdulrahman Bin Faisal University, Dammam 31441, Saudi Arabia; mhajri@iau.edu.sa (M.M.A.); ghayah.a.m@gmail.com (G.A.); ahmed_ms_sa@hotmail.com (A.A.); 2 King Fahad Hospital of the University, Faculty of Medicine, Imam Abdulrahman Bin Faisal University, Dammam 31441, Saudi Arabia; m.alhumaidi.alharbi@gmail.com (M.A.A.); fallmsml@gmail.com (F.A.); bader12dx@gmail.com (B.S.A.); ahmedsz.7117@gmail.com (A.S.A.); aljabbarif@gmail.com (F.A.)

**Keywords:** sickle cell disease, osteomyelitis, arthritis, adult, outcome

## Abstract

**Background/Objectives**: Osteoarticular infections are common complications of sickle cell disease (SCD), often posing significant challenges in diagnosis and management. They primarily affect children: however, the recurrence or emergence of these infections in adults as new complications is well documented. Despite this, there is a notable lack of literature focused on diagnosis and management strategies for adult SCD patients. Our research aims to explore the management challenges in adult SCD patients and to evaluate the outcomes of a selected conservative management approach. **Methods**: The authors conducted a single-center retrospective observational study from January 2018 to December 2022. All adult SCD patients admitted with suspected or confirmed osteoarticular infections were included. Relevant data were meticulously extracted from patients’ hard and electronic medical files. Descriptive statistics were used to present the frequencies and percentages, and suitable statistical analyses were employed to identify specific clinical features and management outcomes in adult patients with SCD. **Results**: Thirty-one patients with osteoarticular infections were included; the majority were males (87.1%) with a mean age of 26.55 years. Long bones were frequently affected, with femurs being the most infected sites (28.1%). Infection recurred in 41.9% of patients. Most patients were managed conservatively (93.5%), primarily with clindamycin and ciprofloxacin, for approximately six weeks, resulting in an excellent cure rate of 96.8%. **Conclusions**: The current study highlights the specific clinical features of osteoarticular infections in adult patients with SCD, identifies radiological findings on Magnetic Resonance Imaging, and suggests a conservative, non-invasive approach for management with excellent outcomes.

## 1. Introduction

Sickle cell disease (SCD) is a group of inherited hemoglobinopathies caused by mutations in the globin gene, producing structurally abnormal hemoglobin. The term SCD encompasses sickle cell anemia (SCA) (Hb SS), hemoglobin S combined with hemoglobin C (Hb SC), hemoglobin S associated with β-thalassemia (Sβ0 Thal and Sβ+ Thal), and other double heterozygous conditions that cause clinical disease.

Despite relatively stable national incidence rates, the global burden of SCD increased from 2000 to 2021, with the total number of newborns diagnosed with SCD rising by 13.7% and the number of individuals living with SCD growing by 41.4%. This increase is primarily attributed to population growth in the Caribbean and sub-Saharan Africa [1]. Sub-Saharan countries in Africa and India are significantly affected by SCD, with an incidence rate of up to 1% among newborns and high childhood mortality rates [2]. In Saudi Arabia, the prevalence of SCD varies, with the Eastern Province and the southwestern region showing the highest rates. While data on prevalence are limited, it is estimated that the sickle cell trait could be found in 2–27% of the Saudi population, with up to 1.4% having sickle cell disease [3].

SCD patients are more susceptible to serious infections, such as pneumonia, bacteremia, urinary tract infections, and musculoskeletal infections, which often manifest as osteomyelitis and septic arthritis, compared to the general population [4,5]. Bone infarctions (osteonecrosis) frequently precede the development of bacterial osteomyelitis in SCD patients. However, differentiating between the two requires advanced imaging, definitive bone biopsy, and cultures. Unfortunately, the availability of such advanced imaging or the ability to identify the causative agent may be limited, especially in resource-limited settings. Therefore, initiating conservative management becomes essential [6].

Most patients with SCD develop osteomyelitis in childhood before ten, with devastating sequelae in some cases [7]. The recurrence or development of osteoarticular infections as a new complication in adulthood is well documented. However, the literature on managing adult SCD patients with osteoarticular infections is notably scarce. This study aims to highlight the challenges of establishing the diagnosis based on clinical and radiological findings and evaluate the effectiveness of a conservative approach applied in an academic tertiary medical center.

## 2. Materials and Methods

### 2.1. Study Design, Settings, and Population

The authors conducted a single-center retrospective observational study from January 2018 through December 2022 at King Fahad Hospital of the University (KFUH), a tertiary medical center with a capacity of over 500 beds located in an SCD-endemic area of the Eastern Province of Saudi Arabia.

The inclusion criteria for patients were:

1. Adults aged 16 years or older.
2. Confirmed SCD diagnosis through hemoglobin electrophoresis.
3. Admitted with a primary diagnosis of osteoarticular infection or admitted for other reasons but confirmed to have an osteoarticular infection.

We excluded SCA carriers (patients with sickle cell trait) and those who did not complete the treatment course or were lost to follow-up for any reason.

### 2.2. Definitions

We have applied operational definitions based on Lew and Waldvogel’s classification of osteomyelitis as acute or chronic [8]. Acute osteomyelitis is defined as osteomyelitis that develops in a short period of up to one month. In contrast, chronic osteomyelitis recurs in the same bone or has a prolonged course for over three months. In addition, osteomyelitis in SCD patients exhibits unique characteristics, particularly in patients who experience multiple episodes of acute osteomyelitis in different bones. Therefore, we have introduced a new definition for “recurrent osteomyelitis”, which applies when a patient experiences recurrent acute osteomyelitis affecting different bones in separate episodes.

Given the lack of definitive criteria for diagnosing osteomyelitis in patients with SCD, osteoarticular infections in our cohort were diagnosed using the following practical diagnostic approach:

1. High clinical suspicion: Persistent fever, localized pain—especially in long bones—along with signs beyond a typical Vaso-occlusive crisis (VCO), such as swelling, redness, warmth, ulceration, discharge, a palpable fluctuant mass, significant effusion, or draining sinus tract. Also, consider a prolonged illness course with symptoms lasting over one week.
2. Supportive laboratory findings: leukocytosis elevated CRP, thrombocytosis, or increased alkaline phosphatase.
3. Positive imaging studies: In plain X-ray (periosteal elevation, lytic or destructive lesions, new bone formation, sequestrum, sclerosis, or medullary infarction) or Magnetic Resonance Imaging (MRI) (soft tissue edema, abscess, or phlegmon, cortical destruction, rim-enhancing fluid collections, sinus tracts, or bone marrow edema with focal fluid collection) findings.
4. Microbiological evidence: Positive blood cultures or positive cultures from bone or joint aspirates or biopsies.

A confirmed case of osteomyelitis implies the presence of at least two of the classical clinical presentations, including fever, prolonged localized pain, swelling, warmth, and tenderness, along with at least two supportive laboratory evidence (leukocytosis, elevated CRP, thrombocytosis, or increased alkaline phosphatase), and at least one of the following MRI findings: bone marrow edema, distracted bone, sinus tracts, or bone abscesses. On the other hand, probable (suspected) osteomyelitis would have less definitive radiological signs of osteomyelitis on MRI, such as periosteal and adjacent soft tissue edema, cortical bone destruction, or bone necrosis, which might be confused with VOC, avascular necrosis as another common complication of SCD.

### 2.3. Data Collection

Data were collected from both paper and electronic patient records. The demographic data includes the patient’s age, gender, and nationality. The clinical data includes the diagnosis as SCA (Homozygous) or other SCD (heterozygous) according to primary hemoglobin electrophoresis results, number of yearly emergency department (ED) visits, number of intensive care unit (ICU) admissions, osteomyelitis episodes, sites of affected bones, comorbidities, symptoms and signs that correlate with osteomyelitis, splenectomy (surgical or functional), onco-carbide therapy, and vaccinations.

The collected laboratory data includes the results of a complete blood count (CBC), liver function tests (LFT), C-reactive protein (CRP), blood cultures, and tissue or synovial culture, if available. The collected radiological data includes the findings of plain X-rays and MRI. Finally, the management data collected includes surgical interventions, the chosen antibiotic combinations, administration route, dosage, and duration of therapy.

### 2.4. Statistical Analysis

The data were collected, cleaned, and then processed using SPSS 26 (Released 2019; IBM Corp., Armonk, NY, USA). Descriptive analysis involved presenting frequency distributions and percentages for categorical variables, including demographic, clinical, imaging, and management variables. Clinical outcomes were also graphed, while routine laboratory findings were reported as mean ± standard deviation and range. All statistical methods were two-tailed. A *p*-value ≤ 0.05 was considered significant.

## 3. Results

### 3.1. Demographic and Clinical Data

Of 36 adult SCD patients diagnosed with osteoarticular infections during the selected study period, 31 were included. Four patients were excluded due to missing data, as they failed to attend follow-up clinics after being discharged. Additionally, one patient was excluded because he did not complete the prescribed antibiotic course, despite experiencing initial improvement, and was discharged against medical advice. The vast majority of the study cohort (87.1%) were males and Saudis (93.5%), with ages ranging from 14 to 49 years (Mean ± SD = 26.55 ± 7.28). According to the primary hemoglobin electrophoresis, 19 (61.3%) patients were homozygous (SCA patients). The remaining were heterozygous and had other SCDs. The most prevalent comorbidities observed were glucose-6-phosphate dehydrogenase (G6PD) deficiency, affecting 60.9% of the patients, and β-thalassemia/α trait, affecting 30.4%. Data related to the number of yearly ED visits, ICU and ward admissions, osteoarticular infection types, clinical characteristics, and onco-carbide therapy were presented in Table 1.

All our patients were diagnosed with either suspected or confirmed osteomyelitis; according to the abovementioned definitions, acute, recurrent, or chronic osteomyelitis was observed in.

**Figure 1:**
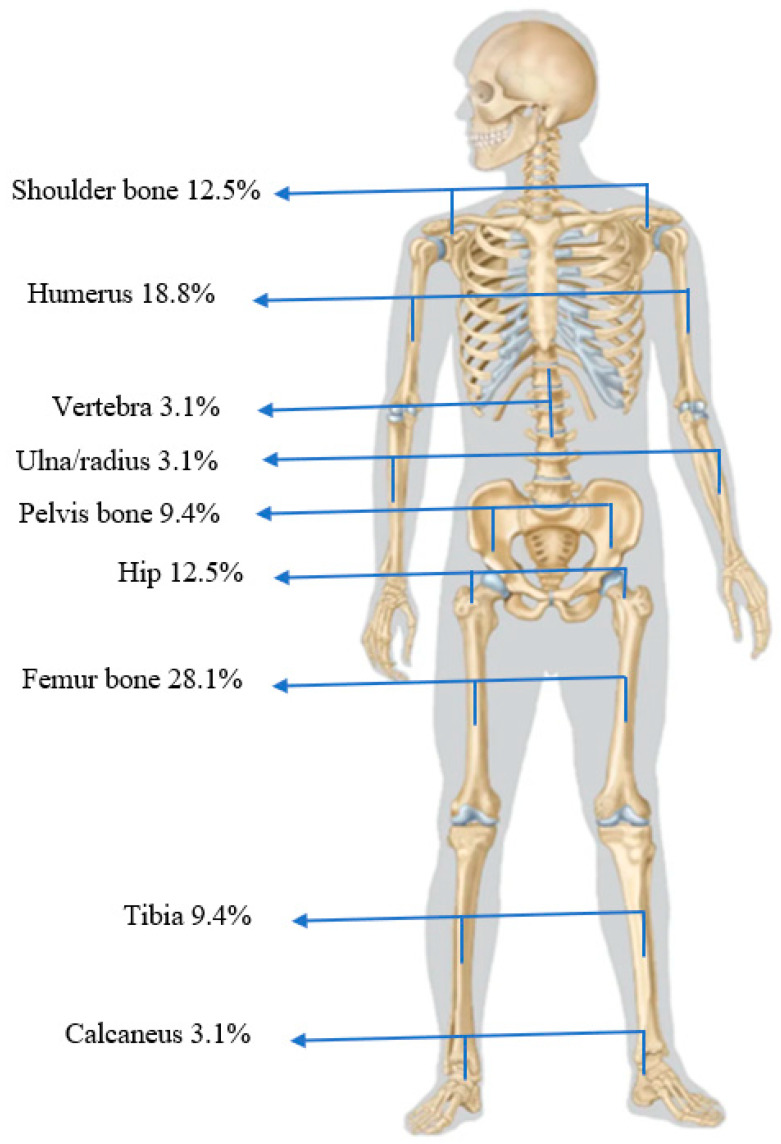
Bones involved in SCD adult patients with Osteoarticular infections.

**Figure 2:**
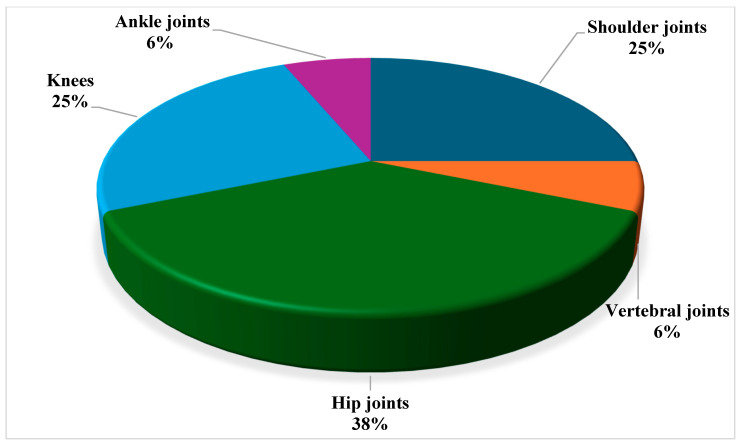
Joints involved in SCD adult patients with Osteoarticular infections.

48.3%, 9.7%, and 41.9%, respectively. The most reported osteomyelitis sites were femurs (28.1%), humerus (18.8%), hips (12.5%), tibias (9.4%), and pelvis (9.4%) (Figure 1). 53.3% of patients demonstrated associated arthritis, with the main joints involved being the hip, shoulder, and knee joints in 37.5%, 32.0%, and 25.0% of cases, respectively (Figure 2).

### 3.2. Laboratory and Microbiological Results

The laboratory data showed, in addition to the typical SCD findings, other abnormalities such as reactive thrombocytosis (399,500 ± 209,100/µL), elevated CRP (13.8 ± 9.7 mg/dL), and increased alkaline phosphatase (157.5 ± 88.5 IU/L), which may support the diagnosis of osteomyelitis (Table 2). On the other hand, microbiological data showed that only 3 of 31 patients (9.7%) had positive blood cultures for *Staphylococcus* species, and 5 of 8 patients (62.5%) had positive tissue/synovial cultures (Mainly Gram-positive; three revealed *Staphylococcus* infections, one *Enterococcus faecalis* and *Bacillus cereus*, and one *Micrococcus lylae*). All cases with positive blood cultures, as well as four out of five positive tissue/synovial cultures, were observed in osteomyelitis-confirmed cases. No courses of antibiotic prophylaxis or *Salmonella* vaccines were administered to any of the involved patients.

### 3.3. Radiological Findings

**Figure 3:**
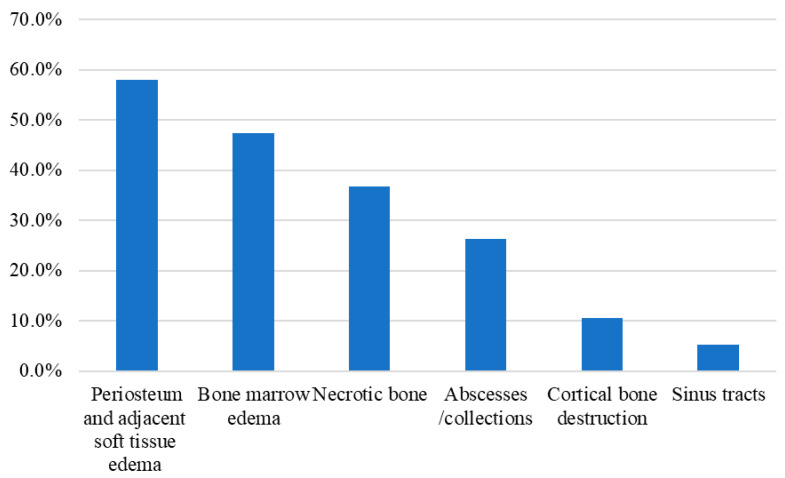
MRI findings of osteomyelitis in the study cohort.

Concerning the radiological findings, the most reported plane X-ray findings were adjacent tissue swelling, periosteal reaction, regional osteolysis, cortical breach, and bone destruction. At the same time, MRI (Figure 3) revealed the presence of periosteum and adjacent soft tissue edema (57.9%), bone marrow edema (47.4%), necrotic bone (36.8%), abscesses/collections (26.3%), cortical bone destruction (10.5%), and sinus tracts (5.3%). 

By comparing confirmed and suspected cases of osteomyelitis, no significant differences were found in demographic variables, frequency of ED visits or hospital admissions, number of osteomyelitis episodes, comorbidities, clinical manifestations, surgical or functional splenectomy, or onco-carbide therapy (Table 3). Additionally, laboratory findings did not reveal statistical significance in relation to confirmed osteomyelitis cases (Table 2).

Moreover, no significant differences existed between confirmed and suspected osteomyelitis, regardless of whether the patient had SCA or another SCD. However, there were significant differences between the suspected and confirmed cases regarding MRI findings; bone marrow edema and necrotic bones were more prevalent in the confirmed cases. In addition, five confirmed cases showed abscesses/collection features, which were not observed in all suspected cases. Nonetheless, this difference was statistically significant (Table 3).

### 3.4. Management and Outcomes

Regarding osteoarticular infection management, all our patients were managed conservatively with antibiotics; however, two patients required surgical interventions (6.5%). The first patient had shoulder osteomyelitis and did not respond to antibiotic therapy, developing an abscess after three weeks of conservative treatment. The patient underwent incision and drainage of the abscess with local bone debridement. The other had extensive pelvic and femoral osteomyelitis, which necessitated aggressive surgical debridement and sequestrectomy, along with dead space management using local antibiotic carriers. Regular weekly follow-up of discharged patients ensured adherence to antibiotic therapy after discharge. All patients received a combination regimen, mainly consisting of clindamycin and ciprofloxacin (71.0%). The average duration of antibiotic therapy was approximately 37 days, resulting in an impressive cure rate of 96.8% (Table 4).

Regarding long-term outcomes, all patients were monitored for at least one year after completing therapy. Three patients (9.7%) experienced recurrent osteomyelitis, as defined above, and were successfully treated. Additionally, one patient who underwent surgery still required walking assistance after 9 months of treatment. All other patients with acute or chronic osteomyelitis had favorable long-term outcomes.

## 4. Discussion

Sickle cell patients experience a range of musculoskeletal complications, including osteoporosis, osteonecrosis, fractures, dactylitis, osteomyelitis, and septic arthritis [9,10].

The main observed bony changes include bone marrow hyperplasia caused by chronic anemia, bone necrosis, and cortical bone thinning, which predispose patients to spontaneous pathological fractures [11]. Developing osteomyelitis frequently complicates the course of SCD, with a reported occurrence rate ranging from 9.2% to 61% [10,12]. The reported local prevalence of bone and joint infections is 17% [13]. The recent study sheds light on clinical features, management challenges, and outcomes of osteoarticular infections in adult SCD patients, presenting a suggested conservative syndromic approach with excellent outcomes.

In addition to recurrent VOC and bone infarctions, observed immunological defense defects predispose osteoarticular infections, such as functional asplenia, impaired phagocytosis, opsonization defect, and decreased complement activity [7,14]. The prevalence of bone infections in patients with SCD is 69 times higher than in the general population [10].

The majority develop osteoarticular infections early in the disease course; male predominance was reported in pediatric patients with a male/female ratio up to 1.8:1 [15,16,17]. However, our study showed that 87.1% of the study population were males with a male/female ratio of 6.8:1. The lifestyle of males compared to females in our population may partially explain this notable gender difference. Osteoarticular infections tend to be recurrent and multifocal; in a 15-year review, Sadat et al. reported 327 episodes in 201 SCD patients, which were multifocal in 23.8% of patients [17]. Our study yielded comparable findings with 49 episodes in 31 adult patients.

The distinction between acute osteomyelitis and other clinical manifestations of SCD is often challenging, as symptoms and signs, laboratory findings, and radiological abnormalities that occur in osteomyelitis are also observed in VOC, infarction, or osteonecrosis [7,18,19].

The predominant clinical manifestations of osteomyelitis reported in SCD pediatric patients are localized pain, swelling, and fever [16,20]. In contrast, our findings showed that only 29.0% of participating adults presented with fever, while localized pain and swelling are still the cardinal clinical manifestations in adulthood (Table 1).

Developing osteoarticular infections at multiple sites was frequently observed in children with SCD and, in many cases, could be symmetrical or affect various foci simultaneously [21,22,23]. The involvement of more than one site in our adult SCD cohort was also high, with 51.6% of patients developing new infection episodes either at the same site (chronic) or at a different site (recurrent). However, the infections were rarely symmetrical or simultaneous.

Regarding the affected bones, the existing literature indicates that no bone can be exempt; however, osteomyelitis primarily affects the long bones [7,21,24]. The tibia was the most frequently reported site of osteomyelitis in studies that included mixed cohorts of SCD patients (children and adults) or only children [4,12,17]. In our cohort, long bones were also frequently affected; however, the femur and humerus were the most impacted bones (Figure 1). 

Routine laboratory tests are never conclusive in diagnosing osteoarticular infections. However, our data revealed reactive thrombocytosis, high CRP, and high alkaline phosphatase, which indicate an inflammatory process and could support the diagnosis of osteoarticular infections.

In our cohort, blood cultures turned positive in only 3 patients (9.7%), which aligns with previous studies showing that isolating causative agents in SCD patients remains difficult and less attainable [6,15]. *Salmonella* and *Staphylococcus* species were the main reported causative agents in the literature [20,25,26,27]. Our institutional antibiograms during the study period revealed that *Salmonella* accounted for 2.8% of all Gram-negative isolates, and *Staphylococcus aureus* accounted for 32.8% of all Gram-positive isolates. Additionally, *Staphylococcus* was the predominant isolated organism through blood, tissue, or synovial cultures; none of the cultures grew *Salmonella*. The predominance of *Staphylococcus* has also been previously reported in sub-Saharan Africa and the Middle East, as well as in published data from Saudi Arabia. In contrast, *Salmonella* species were dominant in Europe and the United States [6,28].

Furthermore, previous studies reveal that *Salmonella* skeletal infections among SCD patients commonly occur in childhood. In contrast, *Staphylococcus* infections more frequently occur in adulthood [23,29,30]. The reported average age of osteoarticular infections caused by *Salmonella* was 6.8 years, compared to 22.1 years for those caused by *S. aureus* [29]. However, Gram-negative organisms other than *Salmonella* have emerged as relevant causative agents of osteomyelitis in SCD patients, such as *Enterobacter cloacae*, *Pseudomonas aeruginosa*, and *Bacteroides* [31].

According to local data from the Eastern Province of Saudi Arabia, Salmonella infections were approximately 18 times higher in SCD patients than in other hospitalized patients [30]. However, *Staphylococcus* species were the main causative agents of osteomyelitis in Saudi SCD patients [28]. Indeed, several factors influence the selection of causative pathogens in patients with SCD. These include the patient’s immune status, flora, and age, as well as the pathogen characteristics, endemicity, and virulence.

Regarding radiological diagnosis, plain X-rays may help establish the diagnosis of an advanced osteoarticular infection. Nonetheless, MRI is considered the preferred imaging modality at any stage of osteomyelitis [32]. However, the difficulty of diagnosing osteomyelitis in SCD patients persists due to considerable overlap with bone infarctions, necrosis, and thrombotic marrow crises [7,16,18,32,33]. Based on clinical manifestations, laboratory results, and the selected MRI findings, we were able to confirm the diagnosis of osteomyelitis in 19 (61.3%) patients. These findings are comparable to the previously reported MRI-based osteomyelitis diagnosis rate of 64.0% [34]. Our data indicate that four MRI findings may serve as diagnostic criteria for osteomyelitis in patients with SCD: bone marrow edema, necrotic bone, sinus tracts, and bone abscesses or collections (Figure 3). The potential of the gadolinium-enhanced MRI technique and scintigraphy using Technetium-99m to improve accuracy is a hopeful prospect [33,35,36]. Unfortunately, scintigraphy and gadolinium-enhanced MRI were not available at our institution.

The management of osteomyelitis in patients with SCD remains controversial. There is no consensus on the best interventions or standardized antimicrobial regimens globally. Variation in pathogen endemicities and the rise of drug resistance hinder the standardization of antibiotic therapy [37].

Surgical interventions, including incision, drainage, and bone drilling, along with parenteral antibiotics administered for six weeks, were recommended, achieving a high success rate of up to 97% [17,27]. However, many of these patients are treated conservatively [20,22,32]. The suggested empirical treatment usually targets *Salmonella* and *Staphylococcus* species [20,21]. Ciprofloxacin and vancomycin were suggested as the initial antibiotic therapy [38]. Data from Saudi Arabia indicated a low prevalence of *Salmonella* and high sensitivity to ciprofloxacin. However, concerns have emerged regarding the development of multidrug-resistant strains [39,40].

Considering that the majority of our patients were diagnosed with acute osteomyelitis and the findings of previous literature, which identified *Salmonella* and *Staphylococcus aureus* as the primary causative agents [4,20,21], and our local microbiological data at KFHU, we chose to manage our cohort conservatively. All patients received antibiotic combinations, primarily clindamycin and ciprofloxacin, for approximately six weeks. This approach yielded an excellent cure rate of 96.8%, offering a promising potential outcome for patients with similar conditions.

The incidence of certain infections, such as influenza and parvovirus B19 infection, decreased during the COVID-19 pandemic period, mainly due to lockdown, reduced travel, and mask-wearing [41,42]. However, we do not observe a significant change in osteomyelitis incidence among adult SCD patients during the pandemic period (18 patients in three years) compared to the pre-endemic period (13 patients in two years). It is important to note that a small study population may limit the ability to draw definitive conclusions.

Finally, the current study has notable limitations, as it is a retrospective observational study with potential gaps in certain data. Additionally, there is no comparison group to assess the effectiveness of the suggested therapy approach against other treatment strategies. Furthermore, the study is confined to a single academic center and does not examine regional and institutional differences in pathogen prevalence and susceptibility. Moreover, the available microbiological data, including antimicrobial sensitivity, were limited, which significantly hampers the generalizability of the study results.

## 5. Conclusions

The current study compares the specific clinical features of osteoarticular infections in adult SCD patients with those in pediatric patients, highlights clinical and radiological criteria to confirm osteomyelitis diagnosis in adult SCD patients, and recommends a conservative, non-invasive syndromic approach for management that yields excellent outcomes. Further studies are needed to establish evidence-based guidelines for the conservative management of these cases.

## Figures and Tables

**Table 1:** Demographic characteristics and clinical variables of adult patients with SCD and osteomyelitis.

Bio-Demographics	N	%
**Gender**		
Male	27	87.1%
Female	4	12.9%
**Age in years (Mean ± SD)**	26.55 ± 7.28	
**Nationality**		
Saudi	29	93.5%
Non-Saudi	2	6.5%
**SCA (homozygous patients)**	19	61.3%
**SCD (heterozygous patients)**	12	38.7%
**Number of yearly ED visits (Mean ± SD)**	5.48 ± 3.67	
**Number of yearly admissions (Mean ± SD)**	2.45 ± 1.50	
**Number of ICU admissions (Mean ± SD)**	1.12 ± 0.78	
**Co-morbidities**		
G6PD deficiency	14	60.9%
β-thalassemia/α trait	10	30.4%
Bronchial asthma	4	17.4%
Avascular necrosis	5	13.0%
Thromboembolic events	3	8.7%
Hypertension	2	8.7%
Pulmonary hypertension	2	8.7%
**Osteoarticular infection type**		
Acute osteomyelitis	15	48.4%
Recurrent (acute) osteomyelitis	3	9.7%
Chronic osteomyelitis	13	41.9%
Joint involvement (arthritis)	16	53.3%
**Clinical manifestations**		
Localized Pain	31	100.0%
Swelling	31	100.0%
Redness	11	35.5%
Hotness	5	16.1%
Fever	9	29.0%
**Surgical splenectomy**	6	19.4%
**Functional splenectomy**	13	41.9%
**Onco-carbide (hydroxyurea)**	20	64.5%

G6PD, Glucose-6-phosphate dehydrogenase; SD, standard deviation.

**Table 2:** Association of laboratory variables with suspected vs. confirmed osteomyelitis.

	Total (N = 31)	Suspected vs. Confirmed OM		*p*-Value
		Suspected OM (N = 12)	Confirmed OM (N = 19)	
Leukocytes count	11.4 ± 5.2	13.27 ± 4.11	10.22 ± 5.63	0.117
Neutrophils %	61.0 ± 14.2	63.93 ± 15.21	56.25 ± 18.60	0.241
Hemoglobin (g/dL)	8.8 ± 1.6	8.79 ± 1.40	8.76 ± 1.84	0.951
Hematocrit	27.5% ± 3.7	28.08 ± 3.04	27.14 ± 4.23	0.507
Platelet count	399.5 ± 209.1	430.17 ± 220.03	380.20 ± 211.47	0.533
Reticulocytes %	6.7% ± 4.1	6.55 ± 3.49	6.85 ± 4.70	0.849
C-reactive protein (mg/dL)	13.8 ± 9.7	12.57 ± 7.67	14.50 ± 11.23	0.607
Total bilirubin (mg/dL)	2.5 ± 2.6	2.41 ± 1.33	2.49 ± 3.20	0.934
Direct bilirubin (mg/dL)	1.6 ± 2.3	1.64 ± 1.44	1.65 ± 2.81	0.992
Alkaline phosphatase (U/L)	157.5 ± 88.5	170.17 ± 99.52	149.53 ± 85.14	0.543
Alanine transaminase (U/L)	32.1 ± 16.1	39.33 ± 17.42	27.47 ± 14.23	0.047 *
Aspartate transferase (U/L)	47.5 ± 32.2	59.17 ± 26.55	40.11 ± 34.66	0.115
Lactate Dehydrogenase (U/L)	456.1 ± 288.2	466.08 ± 242.17	449.79 ± 327.28	0.883

All parameters are presented as Mean ± standard deviation. OM, osteomyelitis. * *p*-Value is significant.

**Table 3:** Association of demographics and clinical variables with suspected vs. confirmed osteomyelitis.

	Suspected vs. Confirmed OM				*p*-Value
	Suspected OM (N = 12)		Confirmed OM (N = 19)		
	No.	%	No.	%	
**Gender**					
Male	11	91.7%	16	84.2%	0.546
Female	1	8.3%	3	15.8%	
**Age in years (Mean ± SD)**	28.3 ± 9.8		25.5 ± 5.4		0.224
**SCA (homozygous patients)**	8	66.7%	11	57.9%	0.625
**SCD (heterozygous patients)**	4	33.3%	8	42.1%	0.625
**Number of yearly ED visits (Mean ± SD)**	6.00 ± 4.82		5.16 ± 2.95		0.550
**Number of yearly admissions (Mean ± SD)**	2.58 ± 1.38		2.37 ± 1.64		0.709
**Number of ICU admissions (Mean ± SD)**	1.17 ± 1.19		0.68 ± 1.11		0.261
**Recurrence of osteoarticular infections**					
One time	10	83.3%	12	63.2%	0.430
Two times	3	16.7%	9	31.6%	
Three times	0	0.0%	1	5.3%	
**Co-morbidities**					
G6PD deficiency	3	25.0%	10	52.6%	0.129
β-thalassemia/α trait	3	25.0%	7	36.8%	0.492
Bronchial asthma	2	16.7%	2	10.5%	0.619
Avascular necrosis	1	8.3%	4	21.1%	0.348
**Clinical manifestations**					
Localized pain	12	100.0%	19	100.0%	-
Swelling	12	100.0%	19	100.0%	-
Redness	6	50.0%	5	26.3%	0.179
Hotness	3	25.0%	2	10.5%	0.286
Fever	5	41.7%	4	21.1%	0.218
**MRI abnormalities**					
Periosteum and adjacent soft tissue edema	4	33.3%	7	36.8%	0.842
Bone marrow edema	0	0.0%	9	47.4%	0.005 *
Cortical bone destruction	0	0.0%	2	10.5%	0.245
Necrotic bone	0	0.0%	7	36.8%	0.017 *
Sinus tracts	0	0.0%	1	5.3%	0.419
Abscesses/collections	0	0.0%	5	26.3%	0.052
**Surgical splenectomy**	3	25.0%	3	15.8%	0.527
**Functional splenectomy**	3	25.0%	10	52.6%	0.130
**Onco-carbide (hydroxyurea)**	6	50.0%	14	73.7%	0.180

* *p*-value is significant, G6PD, Glucose-6-phosphate dehydrogenase; SD, standard deviation.

**Table 4:** Antibiotic combination therapy applied, cure rates, and therapy duration.

Antibiotic Combination Therapy	Treated Patients N (%)	Cured Patients N (%)
Clindamycin + Ciprofloxacin	22 (71.0%)	21 (95.5%)
Clindamycin + Piperacillin/tazobactam	5 (16.1%)	5 (100%)
Clindamycin + Ceftriaxone	2 (6.5%)	2 (100%)
Cefazolin + Ciprofloxacin	2 (6.5%)	2 (100%)
**Total**	31 (100%)	30 (96.8%)
Antibiotic therapy duration in days *	36.7 ± 21.2	

* Mean ± standard deviation.

## Data Availability

The data are available upon reasonable written requests to the corresponding author, including a research proposal and a data-sharing agreement.

## Abbreviations

CBC	Complete Blood Count
CRP	C-reactive protein
ED	Emergency department
G6PD	Glucose-6-phosphate dehydrogenase
ICU	Intensive Care Unit
LFT	Liver Function Tests
MRI	Magnetic Resonance Imaging
OM	Osteomyelitis
SCA	Sickle cell Anemia
SCD	Sickle cell disease
VOC	Vaso-occlusive crisis
